# Adsorption of Different Pollutants by Using Microplastic with Different Influencing Factors and Mechanisms in Wastewater: A Review

**DOI:** 10.3390/nano12132256

**Published:** 2022-06-30

**Authors:** Meng Zhao, Lei Huang, Samuel Raj Babu Arulmani, Jia Yan, Lirong Wu, Tao Wu, Hongguo Zhang, Tangfu Xiao

**Affiliations:** 1 School of Environmental Science and Engineering, Guangzhou University, Guangzhou 510006, China; ymzguangzhou@163.com (M.Z.); huanglei@gzhu.edu.cn (L.H.); jarleumas@ymail.com (S.R.B.A.); jiayan@gzhu.edu.cn (J.Y.); a469949085@163.com (L.W.); wutaogzhu@163.com (T.W.); tfxiao@gzhu.edu.cn (T.X.); 2 Guangzhou University-Linköping University Research Center on Urban Sustainable Development, Guangzhou University, Guangzhou 510006, China; 3 State Key Laboratory of Geohazard Prevention and Geoenvironment Protection, Chengdu University of Technology, Chengdu 610059, China

**Keywords:** organic pollutants, inorganic pollutants, adsorption

## Abstract

The studies on microplastics are significant in the world. According to the literature, microplastics have greatly specific surface areas, indicating high adsorption capacities for highly toxic pollutants in aquatic and soil environments, and these could be used as adsorbents. The influencing factors of microplastic adsorption, classification of microplastics, and adsorption mechanisms using microplastics for adsorbing organic, inorganic, and mixed pollutants are summarized in the paper. Furthermore, the influence of pH, temperature, functional groups, aging, and other factors related to the adsorption performances of plastics are discussed in detail. We found that microplastics have greater advantages in efficient adsorption performance and cost-effectiveness. In this paper, the adsorptions of pollutants by microplastics and their performance is proposed, which provides significant guidance for future research in this field.

## 1. Introduction

With the increasingly serious environmental pollution, sewage treatment has become a worldwide research hotspot, and the technology of microplastics adsorbing harmful substances to purify sewage has become a prominent choice [1]. Adsorption technology is developing rapidly, and the development of large-scale and sustainable hybrid technologies can overcome the shortcomings of traditional technologies. Microplastics have the advantages of large surface area and low cost [2], thus, leading to applications in the field of sewage treatment.

The American Association of Marine Conservation Scientists defines plastic as a synthetic water-insoluble polymer. According to the relevant literature, plastics are divided into thermosetting and thermoplastic plastics. The high abundance of microplastics (MPs) can be attributed to three factors: first, human activity, such as tourism waste, or waste nets from fishing can degrade to produce MPs [3]; second, industrial supplies, such as beverage cans, plastic bags, and plastic water cans. 

Severe acute respiratory syndrome coronavirus 2 (SARS-CoV-2) is the virus responsible for the COVID-19 pandemic. Since it emerged, the disease has led to high mortality and morbidity [4]. In the process of testing for the SARS-CoV-2, a large of plastic products flowed into the environment. In addition, there are some modern products, such as the increased of using cosmetics [5] and textiles [3]. 

MPs are emerging pollutants that have been shown to provide toxic contaminants [6]. For example, the pathways by which the environment or its inherent toxic monomers and additives enter the aquatic food web were MPs [7]. MPs can enter agroecosystems with toxic effects [8] but also enter organisms [9] to cause cellular internalization and affect intracellular target organelles [10]. Moreover, there are many difficulties in fully solving nano plastics due to the limited methodologies for selectively recognizing nano plastics [11].

Traditional sewage treatment technology is complex in operation; has high costs, slow adsorption, limited capacity, and high regeneration costs; and may cause the risk of secondary pollution. Thus, finding a cheap and simple treatment method has become an urgent problem. Based on the principle of green environmental protection, waste utilization is the best way. Microplastics have been used to make electrospun micro/nanofibers (in the waste recycling) [12], PET nanoparticles (in the biological systems) [13], and antimicrobial coatings (in the healthcare sector) [14]. 

The unique structures of microplastics with benzene rings or carbon chains make them a good material choice for sewage treatment. As microplastics also contain rich functional groups, they have a strong affinity for pollutants under the action of chemical bonds, such as hydrogen bonds and van der Waals forces, and are suitable for sewage treatment [15]. Although pollutants are adsorbed on plastics and cause certain harm to the environment [16], we can achieve simultaneous removal of pollutants and microplastics through this method, which is a good water source treatment method [17].

Microplastics have attracted extensive attention due to their large specific surface area and stable properties [18]. Sewage treatment by microplastics mainly depends on adsorption, and the advantage of microplastics in the specific surface area greatly increases the contact area with pollutants. In addition, the resin carbon structure is stable and easy to separate. Therefore, after treating sewage, plastic can be separated from sewage by centrifugation or sedimentation. 

Under the influence of the environment, light, weathering [19], breakage, corrosion, grafting, abrasion, single loading, aging [20], and other effects will not only hinder the treatment of sewage by microplastics but also increase the specific surface area of microplastics. When microplastics react with pollutants in the environment, modified microplastics can show strong adsorption performance for some contaminants under this influence [21]. This paper reviews the classification of microplastics and the adsorption mechanism of microplastics on organic and inorganic pollutants. In addition, the activities of microplastics in nature and their treatment methods and principles are briefly introduced. Hence, we provide new ideas for utilizing microplastics with contaminants.

## 2. Activity of Microplastics in the Environment

### 2.1. Activities of Microplastics in Soil

**Figure 1:**
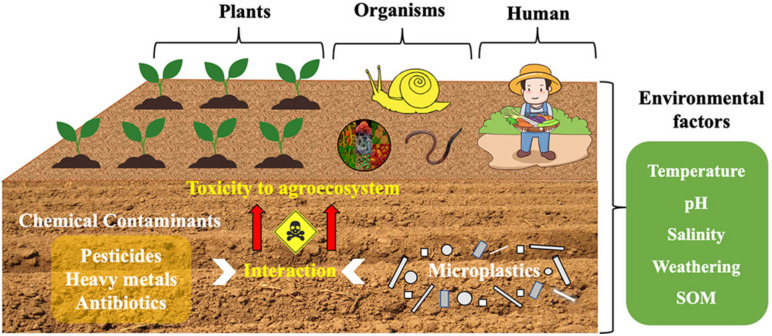
Interaction diagram between microplastics and pollutants in soil [22].

Although the activity of microplastics in the soil environment is limited, the amount of microplastics in the soil cannot be ignored. Most human activity is on land, which contributes to the accumulation of microplastics from landfills, fermentation and composting of organic fertilizers, and the use of tires. Under the influence of temperature, pH, and other factors, as shown in Figure 1, microplastics can still interact with pollutants in the soil environment [22]. Microplastics in the land mainly interacted with pollutants directly to realize the treatment of pollutants. Of course, the indirect effects of microplastics also affect soil properties [23], such as soil MPs, can provide sorption sites for soil microorganisms, establish unique microbial communities and influence the ecological role of soil microorganisms [24].

### 2.2. Activities of Microplastics in Water

**Figure 2:**
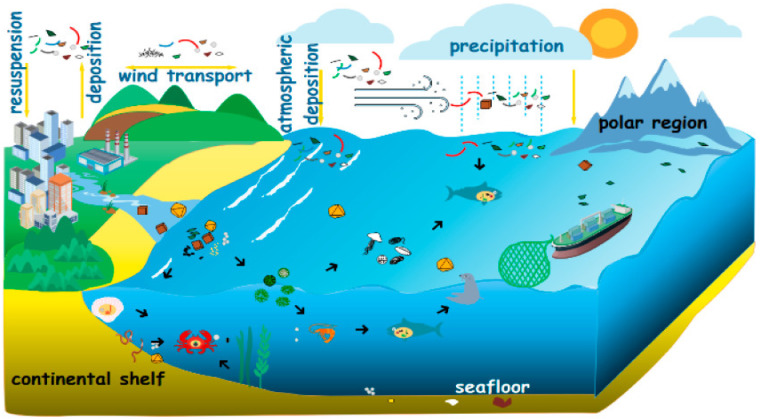
Origin and cycle process of microplastics [25].

At present, microplastics have been stored in large amounts and have frequent activities in the marine environment. The main phenomenon is the suspended atmosphere [25] as shown in Figure 2. Global marine plastic pollution is derived mainly from the input of vast amounts of land-based plastic waste [26]. Wastewater treatment plants (WWTPs) are known to be one of the main and most important sources of microplastic discharged into the environment [27]. 

The investigation found that a secondary wastewater treatment work (WWTW) (population equivalent 650,000) was releasing 65 million microplastics into the receiving water every day [28]. The abundance of MPs in the influents and effluents of domestic WWTPs was 18–890 and 6–26 n·L^−1^. No significant differences in MP abundance were demonstrated among effluents or wastewater from domestic, industrial, agricultural, and aquacultural sources, indicating they were all potential sources of MP pollution [29].

**Figure 3:**
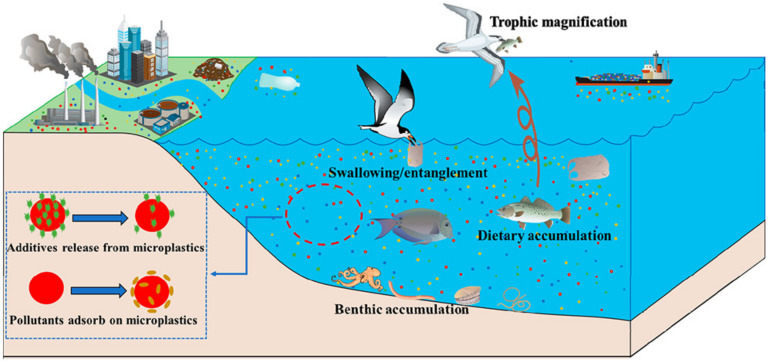
Photodegradation of microplastics and their activities in water environments [30].

MPs release chemical additives during photodegradation [30] as shown in Figure 3. Microplastics deposited at the bottom of water, such as rivers, were easily swallowed and absorbed by aquatic animals and then circulated in the food chain superposition step by step. As a result, bioaccumulation was constantly amplified [31], such as microplastics used as anthracene carriers in medaka [32] and polyvinyl chloride (PVC) used as carriers of heavy metals [33]. 

Some contaminants also exhibited competitive behavior on the surface of microplastics, as DDT interferes with the adsorption of phenylalanine plastics [34]. To date, many kinds of microplastics have been found in the human body. Microplastics can easily combine with various additives and other pollutants in the water environment; however, the discovery that they can be used as carriers of harmful substances [35] brings hope to sewage treatment [36]. In waters, microplastics can not only interact with pollutants but also treat pollutants by winding, adsorption, and other methods. Similar laws inevitably exist in marine, river, lake, and other water environments.

## 3. Classification of Microplastics

Microplastic fragments are formed by the decomposition of larger plastic particles using photolysis, thermal oxidation, thermal degradation, and biodegradation as shown in Figure S1 [37]. Microplastics can be divided into three aspects: (i) polycarbonate polyurethane (PCU) fragments are found in foam plastics. (ii) Polypropylene (PP) and polyethylene (PE) + PP [38] films are found in boat paints. (iii) PET and PES are found in fishing nets and lines and textile production [39] (clothing, wool, and blankets) [40]. The classification of microplastics is determined by their physical and chemical properties and can be divided into five characteristics: their shape, polymer, color, source, and particle size [40] as shown in Table S1.

Shape: The main categories of microplastics by shape are microfibers or plastic fibers, followed by fragments, films, and particles. Microplastics are found in surface water and sediment, and beads make up only a small fraction of the types of microplastics [41].

Polymers: Polymers are identified as polypropylene, polyethylene, and polystyrene [42]. The most significant proportion of microplastic polymer particles is composed of polyethylene [42], followed by polyethylene terephthalate and polyacrylamide. The sediment is dominated by polypropylene. PE and polypropylene (PP) are the most abundant contaminants globally, followed by polymers, such as polyethylene terephthalate, polystyrene, and polyamide.

Grades: In the environment, microplastics are classified as primary plastics and secondary plastics according to their source. Primary microplastics include plastic particles manufactured as precursors for personal care products and other industrial uses [43]. Secondary microplastics are created through photolysis and hydraulic friction, which are found in plastic waste, such as fragments, scales, fibers, and particles, such as plastic bottles, plastic bags, and synthetic textile fibers, which are discarded manually. Second, microplastics are larger plastic fragments that are degraded by using (e.g., fibers released from washing clothing or textiles), waste management, or fragments of larger plastics in the natural environment (e.g., fibers released in water and water environments).

Microplastics in the environment have a large base, many types, and complex properties. In the natural environment, microplastics constantly change their forms and structures with the action of nature, and naturally, they have different properties, making their use more flexible in modern conditions [41].

## 4. Classification of Pollutants

There are many kinds of pollutants in complex forms. According to the literature [44], they can be divided into organic pollutants [2] and inorganic pollutants; however, some pollutants do not belong to the first two categories, and thus an additional pollutant is added. The primary organic pollutants are carbon chains and carbon rings. In addition, it contains acids, such as acetic acid, formic acid, and other carbon chains that have acid groups. There are also benzene rings such as phenanthrene, nitrobenzene, and naphthalene. Inorganic pollutants are mostly salts [45]. 

These substances in the water generally cannot maintain the original compound form, often through ionization hydrolysis and other effects in the form of ions free in the water, the most pollutants are metal ions. In addition to ions, there were a small number of metals that can maintain their original form in water, such as mercury and other metals with relatively stable physical and chemical properties. In addition to the common types of pollutants, some fibers, such as fiber carbon, fiber metal materials, etc., can also be classified as inorganic pollutants. Other pollutants are mixtures, such as oils and some medicines. As these substances can automatically decompose and recombine to form new substances under the action of temperature, it is impossible to obtain clear statistics on their content and composition. They are composite pollutants; therefore, they are classified separately.

## 5. Factors Affecting the Adsorption of Microplastics

**Figure 4:**
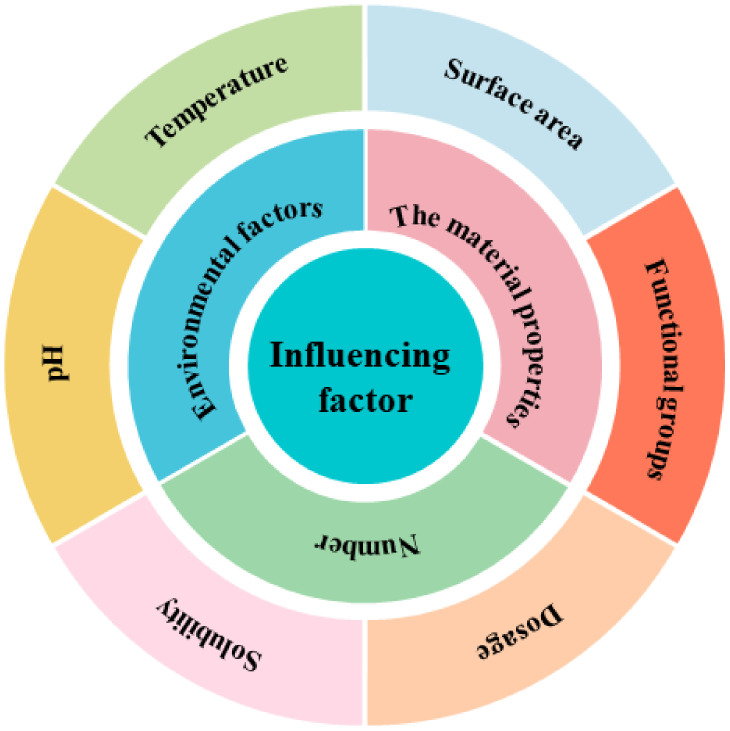
Different types of influencing factors in the process of adsorbing pollutants by microplastics.

There are many types of pollutants, and correspondingly, there are many types of microplastic adsorbents used to treat sewage. The literature review on the adsorption of plastics on various pollutants found that the factors influencing the adsorption of plastics on pollutants include many aspects. Figure 4 shows the adsorption of microplastics on contaminants and their influencing factors. Table 1, Table 2 and Table 3 briefly summarize the adsorption of various pollutants by different microplastics and their influencing factors: (i) the adsorption of organic pollutants by plastics as shown in Table 1; (ii) the adsorption of plastics to inorganic pollutants as shown in Table 2; and (iii) the adsorption of plastics to mixed pollutants as shown in Table 3.

The results were different under different influencing factors. For example, microplastics, heavy metals, and pollutants may form complexes, which exhibited different properties under the influence of atomic valence, atomic size, surface texture [46], and functional groups [47]. Of course, the adsorption effect was different for different adsorbents. Biochar may be an excellent adsorbent for repairing high-concentration NH_4_^+^ wastewater. The interaction between biochar and PE can change the surface properties of the adsorbent [48]. In addition, the same influencing factors, such as pH, temperature, and functional groups, also had different phenomena in various adsorption experiments [49].

### 5.1. Effect of pH

In the process of microplastic adsorption of pollutants, pH was the most significant influencing factor. It had the most crucial effect shown in the adsorption of organic pollutants by microplastics. One of the most important aspects was the effect of pH on the adsorption properties of microplastics, such as microplastics for sulfamethoxazole [41] and three triazole fungicides [50]. There were also some other organic pollutants or compounds adsorbed, and the amount of adsorption increased as the pH value increased.

Of course, the pH value also inhibited the adsorption capacity of microplastics, such as the adsorption capacity of 2,4-hexadiene (sorbic acid) [51], adsorption of perfluorooctane sulfonic acid on polyethylene and polystyrene [52], and adsorption of phenol on polymer resin decreased with increasing pH value [53].

In addition, the inhibition effect of pH value was also reflected in the adsorption of inorganic pollutants by microplastics. Under the influence of pH value, folate-polyaniline hybrid hydrogels tend to adsorb anionic dyes, and a low pH value was more conducive to improving the adsorption capacity of folate-polyaniline hybrid hydrogels [54]. Under basic conditions, iminodiacetic acid chelate resin can form a complex with metal ions [55]. However, alkaline conditions can sometimes produce fatal obstacles to the adsorption of plastics. For instance, the adsorption of bisphenol by polythioctylic acid-based coagulants can directly decrease the adsorption efficiency from 90% to 10% under alkaline conditions [55]. 

In the study of the adsorption of Cr(III) and Fe(III) by modified polyethyleneimine, the alkaline state would precipitate Cr(III) and Fe(III). In contrast, under acidic conditions, modified polyethyleneimine combines more easily with hydrogen ions, and the most favorable condition was a pH of 5 [56]. In addition, sulfamethoxazole mainly existed in the form of anions after ionization in an aqueous solution, and a suitable pH value was conducive to enhance the adsorption affinity of microplastics for organic pollutants [34]. When the pH was 5, the adsorption electrostatic repulsion of nano and microplastics were the lowest.

The effect of microplastic pH on pollutant adsorption did not follow the law of increasing or decreasing. In the case of inorganic pollutants, the impact of adsorption will be the highest under the influence of pH value. This was because pH can affect the group, hydrolysis, and ionization of substances in the solution and jeopardize the adsorption performance [31].

In general, microplastics had an optimal range for the adsorption of metal ion pollutants. The maximum adsorption value would appear in the adsorption process of styrene-DVB resin for homocarboxylic acid. For example, in a weakly acidic environment with a pH value of 4–6, poly (ethylimide) adsorbents were effective in decontamination [57]. The effect of pH on removing hexavalent chromium was carried out in the pH range of 2–9 [54]. When the pH was 5, the adsorption capacity of Cd and Ni ions on polystyrene nanofibers reached the highest value [58]. The most favorable adsorption conditions of polyacrylonitrile-2-aminothiazole resin for mercury, cadmium, lead, copper, zinc, and nickel are 2.50–6.50 [59]. 

When the pH was 5.95, D152 resin adsorbed cadmium(II). For the adsorption of cadmium and nickel ions on polystyrene nanofibers, the adsorption of Er(III) on acrylic grafted polytetrafluoroethylene fiber was the highest [60]. The positive charge of polystyrene microplastics was favorable for the attraction of HA and FA when the pH was less than 2.75. At pH 5, IDA-chelating resin adsorption of bivalent heavy metal ions follows the same principle. In the pH range of 5–9, the content of Cd adsorbed on MPs first increased and then decreased. 

The adsorption effect of IRN77 cation exchange resin on Co(II), Cr(III), and Ni(II) was the best at the pH of the original solution [61], and the adsorption of polyacrylonitrile-2-amino-2-thiazoline resin for precious metal ions [62], adsorption treatment of Cu(II) and Pb(II) with amine and sulfur-chelating resin was similar [63]. In the adsorption of Rh(III), Ru(IV), Pd(II), and Ir(IV) by polyacrylonitrile-thiosemicarbamide resin, a high pH was beneficial to the adsorption of Pd(II) [64]. 

When the pH was 5.8, the most suitable amine chelating resin for Cu(II) and Pb(II) absorption, if the pH was too low, amino groups were accessible to protons. When the pH was too high, metal ions easily precipitate by plugging the pore size. Moreover, it was possible to treat some metal ions in water when the pH was too high, such as the adsorption of copper by microplastics [65]. However, Cu(OH)_2_ precipitation was generated instead of adsorption.

### 5.2. Effects of Particle Size

Microplastics with small particle sizes have a larger adsorption capacity because the specific surface area of plastics with small particle sizes is more prominent, which increases their contact area with pollutants, thus, significantly improving the adsorption performance of plastics. Weathering also increased the specific surface area of plastics, changed the particle size of microplastics, and produces oxygen groups, which would increase their polarity, charge, roughness [66], and porosity [49].

In addition, the same principle applies to crystallinity, with lower crystallinity polymers accumulating more contaminants. In the adsorption experiment of trinitrophenol and TCEP with polyethylene and polyvinyl chloride microplastics, plastic with a small particle size was better. The adsorption of phenanthrene on microplastics and medium plastics followed the same principle, and phenanthrene on microplastics had a more substantial adsorption capacity [67]. In conclusion, adsorbents with larger surface area had a larger adsorption capacity. Table S2 summarizes the surface area of different adsorbents.

### 5.3. Effects of Temperature

In the experiment of ion adsorption by microplastics, the temperature was a critical factor affecting the reaction result. In addition, hydrolysis and ionization can be promoted at high temperatures, and pollutants can be pyrolyzed at high temperatures to improve the sewage treatment capacity of microplastics. For the adsorption of mercury from coal and waste PVC, the adsorption efficiency of mercury increased from 600 to 800 °C. Similar to polystyrene sphere macromolecules with a positive charge, high-temperature polystyrene sphere macromolecules with intense movement can fully collided with anions and attracted each other to adsorb more anionic dyes. In addition, the adsorption of Cr(III) and Fe(III) on modified polyethyleneimine had similar results [68].

As the temperature increases, the adsorption performance of microplastics will also improve, such as the adsorption of cadmium(II) by D152 resin [60], adsorption of Er(III) on acrylic grafted polytetrafluoroethylene fiber [60], adsorption of metal ions, such as Cu(II), by cross-linked polystyrene supported low-generation diethanolamine dendrimers [69] and adsorption of precious metal ions on polyacrylonitrile-2-amino-2-thiazoline resin [62]. Moreover, the adsorption efficiency of polythioctylic acid-based coagulants for organic pollutants, such as bisphenol, decreased by approximately 10% when the temperature dropped from 330 to 277 K [55]. Although, according to various data, the adsorption effect of the grafted PTFE fiber on Cu(II) increased with increasing temperature [70], these spontaneous reactions and endothermic reactions under high-temperature conditions can have a good adsorption effect.

Subsequently, high temperatures were not always the supreme effect. For example, in the adsorption of TnBP and TCEP by polyethylene, the surface tension and van der Waals force of the organic light-emitting materials decreased at high temperatures, which hindered the adsorption performance. In addition, some exothermic reactions, such as the adsorption of As(III) by polytetrafluoroethylene (PTFE) microplastic particles, had an inhibiting effect on spontaneous adsorption [71].

### 5.4. Effects of Salinity 

Salinity also played a specific role in the adsorption process of microplastics, such as the treatment of aromatic amines by polyethylene microplastics. With increasing salinity, the adsorption efficiency gradually increased [20]. Moreover, high salinity in water inhibited the release of carbon nanotubes and photodegradation, and the adsorption properties of microplastics were affected to a certain extent. In the experiment of removing heavy metal ions, coexisting sodium ions grabbed adsorption sites of heavy metals in a short time, thus, reducing the removal efficiency of heavy metals [72].

### 5.5. Effects of Functional Groups

Functional groups are also an essential factor that affects the adsorption of microplastics. The existence of functional groups endowed materials with different properties. As these functional groups showed unique properties, they exhibited excellent adsorption effects on pollutants. In addition to the functional groups of the resin itself, aging [73] and weathering yielded more surface area and produced all kinds of functional groups. This increased their polarity, charge, roughness, and porosity to a certain extent, which affected the adsorption capacity of microplastics [74]. It was proven that the adsorption capacity of PE for chlortetracycline and amoxicillin increased by 1.08–14.24 times after aging [75]. Furthermore, different functional groups may produce different results in acid–base, hydrogen bonding, complexation, etc.

Functional groups in the adsorption of microplastics generally promoted the experiment. For example, in the adsorption experiment of resin for phenol, the amine group on the resin, quaternary amine, primary amine, and secondary amine groups were conducive to the adsorption of resin for phenol, while the tertiary amine group is not [76]. The tert-butyl ester group in glass fiber-reinforced plastics also promoted the adsorption of polyacrylic acid [77]. When coal and waste polyvinyl chloride adsorbed mercury, the carbon chloride functional group could be converted to ionic chloride to realize the adsorption of mercury [78]. 

Calcium ion polymers can effectively adsorb organic micropollutants because of the π-rich network in porous polycalixarenes [79]. Similar to the adsorption of mercury by polyethyleneimine-modified porous cellulose carriers, the adsorption of metal pollutants by microplastics was free from the influence of pH value due to the action of the amine group [80]. Polyacrylonitrile-thiosemicarbazone resin contained triazolthione functional groups, Pd(II) adsorption required one active functional group, while Ru(IV) and Ir(IV) adsorption required two functional groups [64].

However, under exceptional circumstances, the functional groups inhibited the adsorption of microplastics. For example, alcohol groups in cellulose can be acetyl, which changed the structure of cellulose, and cellulose triacetate is more acetyl than cellulose acetate, which reduced its ability to adsorb 2,4-hexadiene (sorbic acid) [51]. Furthermore, with the increasing in the polarity of aromatic amines, the adsorption of polyethylene to aromatic amines was hindered [20]. In contrast to the adsorption experiment on polyethylene and nylon fibers, there was no obstruction of hydrophilic group amide, and polyethylene fiber had a decisive advantage in the adsorption capacity and adsorption speed; however, the other structure of the resin was unable to avoid the effect of inhibition [81]. 

In addition, in the adsorption experiment of Cu(II) by cross-linked polystyrene-supported low-generation diethanolamine dendritic polymer, too many functional groups caused a large area of blockage, and Cu(II) could not adsorb well on the resin [69]. To a certain extent, the adsorption efficiency of coal and waste PVC on mercury gradually increased with increasing PVC content. However, with the addition of abundant PVC, the adsorption efficiency of coal and waste PVC on mercury decreased because too much PVC easily blocked the pore size [78].

### 5.6. Effects of Ionic Strength

Ionic strength plays a small role in the adsorption experiments of microplastics; for example, the adsorption of pyrazystole and dioxystole could promote the experiment; however, for others, it had no similar effect [82]. In addition, due to the salting-out impact, a high salt concentration was more conducive to the adsorption of DPA molecules by microplastics [20]. Moreover, the presence of salt ions in the adsorption of triazole fungicides on polystyrene resulted in the highest adsorption capacity [50].

### 5.7. Effects of Surfactants

In resin adsorption experiments of pollutants, due to their hydrophobicity, the resin for pollutant adsorption efficiency was not high, and the solubility of a surfactant could change the resin. The adsorption performance of the resin, the most representative, was silica, which could adhere to the surface of organic matter or inorganic matter and be soluble in the inorganic or organic matter [83]. The adsorption of cationic methyl bromide on pure polyvinyl chloride was enhanced by mucopolysaccharides [84], which was a typical example of enhanced adsorption of ionic pollutants by surfactants. In addition to sewage treatment, surfactants were also widely used in facial, hair, and clothing cleaning work.

### 5.8. Effects of Solubility, Concentration and Dosage

The different solubilities of pollutants produced different adsorption results. For example, the results of the adsorption experiment on chloromethane by plastic indicated that the adsorption amount of trichloromethane by plastic was greater than the adsorption amount of carbon tetrachloride, which is the reason for the solubility [85], and the adsorption of lead and cadmium in intertidal sediments by facial scrub beads [86]. 

In the adsorption of 2,4,6-nitrotoluene by functionalized polystyrene nanospheres, the adsorption amount initially increased and then flattened because the high solubility of KH-570 obstructs the phosphorus–phosphorus accumulation interaction between the adsorbent and pollutant [87]. However, hydrophobic substances were more easily removed from an aqueous solution than hydrophilic substances, with different solubility and adsorption results [85].

In different experiments, the concentration altered the effects on the adsorption process of microplastics. As in the adsorption of three triazole fungicides by polystyrene microplastics, the concentration promoted the adsorption capacity of polystyrene [50]. Polystyrene can enhance the migration of nonpolar (pyrene) and weakly polar (2,2,4,4-tetrabromodiphenyl ether) compounds in saturated soils. However, in the adsorption of 5-sodium disulfoisphthalic acid by acrylate polymer YWB-7 resin, the methanol content had a hindrance effect on the performance of the adsorbent [88].

High levels of microplastics can absorb more pollutants. For example, with the increase in polystyrene nanofiber sorbent, the adsorption efficiency of cadmium and nickel ions can reach more than 90% [58]. The IRN-77 cation exchange resin for Co(II), Ni(II), and Cr(III), three kinds of ion adsorption efficiencies, is very high, and the increase in IRN-77 cation exchange resin dosage can reach 100% [89]. The same effect was observed with modified polyethyleneimine Cr(III) and Fe(III) adsorption [56]. 

The adsorption of Co(II), Cr(III), and Ni(II) on IRN-77 cation exchange resin followed the same principle [61]. The adsorption efficiency of phenanthrene and its mono hydroxyl derivatives increased with the amount of adsorbent [90]. In addition, some reagents can also affect the adsorption efficiency of microplastics. For example, in the adsorption of 5-sodium disulfoisylphthalic acid by acrylate polymer YWB-7 resin, the amount of methanol can seriously hinder the efficiency of the adsorbent [88].

On the other hand, the pore size of the adsorbent also followed the same principle, such as poly(ethylimide) adsorption of lead and mercury. The larger pore size of the adsorbent can provide better adsorption effect [57]. Thus, the difference in the amount of pollutants adsorbed by these plastics was due to the influence of solubility, concentration, and dosage on the adsorption performance of microplastics.

### 5.9. Effects of Adsorption Selectivity

Adsorption selectivity refers to the excellent adsorption performance of microplastics for some pollutants in the process of treating mixed contaminants. For example, polyaniline hybrid hydrogels showed high selective adsorption capacity for chromium(VI), eosin yellow, rose red, methyl orange, and other anionic pollutants but showed low adsorption capacity for mercury(II), lead(II), rhodamine B, bismuthite Brannia methyl blue, and neutral red [54]. 

On the other hand, polystyrene-deg-3-ap, polystyrene-TeG-3-AP 3-aminopyrine, and hydrophilic spacer arm chelating resin for Hg(II), Ag(I), Fe(III), Pb(II), Co(II), Cu(II), Ni(II), Cd(II), Zn(II) plasma, and Hg(II) showed a strong adsorption capacity [91]. In addition, phenanthrene had stronger adsorption on microplastics than did 1-nitronaphthalene. Polarity enhanced the adsorption of 1-nitronaphthalene on microplastics [92].

**Table 1:** Adsorption of organic pollutants.

Adsorbent	Size Range	Pollutants	Influencing Factors	Reference
plastic cellulose acetate	---	2,4-diallyl (sorbic acid)	pH	[51]
PE, PS, PVC	---	PFOS, FOSA	pH	[52]
polymer resin	---	phenol	pH	[53]
polylipoic acid ester-base coagulant	0.40–0.56 and 0.15–0.30 mm	organic pollutants (pops)	temperature, pH	[55]
HDPE, PS, LDPE, PVC, SSA	---	the Philippines, Nitrobenzene and naphthalene	particle size, crystallinity,	[67]
anion exchange resin	---	phenol	amino	[76]
glass fiber reinforced plastic	---	polyacrylic acid	tert-butyl ester groups	[77]
porous polycarboxarene	---	organic micropollutants	π	[79]
the original rusty water microplastic	0.2 mm	ions, organic pollutants	the surfactant	[84]
polyethylene, neoprene, polyvinyl chloride and polyurethane foam	12 mm	chlorinated methane	chlorinated methane solubility, concentration	[85]
functionalized polystyrene nano ball	---	2,4,6-trinitrotoluene (TNT)	the dosage of KH-570	[87]
acrylate polymer YWB-7 resin	0.4–0.6 mm	5–2 sulfo sodium isophthalic acid	methanol content	[88]
micro polyvinyl chloride (PVC) plastic	80–210 μm	Fe and single hydroxyl derivatives	adsorbent dosage	[90]
PA, PVC, PET	---	sulfanilamide	UV-irradiation, temperature	[93]
polyethylene and polyvinyl chloride (PVC) plastic	1–5, 0.425–1, 0.125–0.425 and 0.045–0.125 mm	san zhang butyl ester phosphate and phosphate (2-ethyl chloride)	particle size	[94]
polystyrene	80.4 ± 7.9 nm	organic pollutants (pops)	the concentration of	[95]
biological membrane reinforced plastic microfiber	2–3 mm	perfluorinated octane sulfonic acid (PFOS)	aging	[96]
PE, PS, PA, and PVC	152.53 ± 57.92, 168.55 ± 57.50, 109.44 ± 44.53, and 57.64 ± 26.50 μm	nonpolar organic compounds	adsorbent performance	[97]
PS and PP	3.5 mm in length +2.2 mm wide and 3–5 mm	fuel aromatics and ether	aging	[98]
polystyrene	29 μm	nonionic organic compounds	functional groups	[99]

**Table 2:** Adsorption of inorganic pollutants.

Adsorbent	Surface Area	Pollutants	Influencing Factors	Reference
polystyrene (MP_S_)	---	nanometer oxide (CeNPs)	heavy metals (HM)	[46]
iminodiacetic acid chelating resin	0.40–0.56 and 0.15–0.30 mm	Sc(III), Y(III), La(III), Fe(III), Al(III), Ga(III), In(III)	pH	[55]
modification of polyethyleneimine	0.15 and 0.075 mm	Cr(III), Fe(III)	pH, adsorbent dosage, temperature	[56]
polyethylimine	---	Pb and Hg	the aperture	[57]
polystyrene nanofibers	---	Cd, Ni	pH, adsorbent dosage	[58]
polyacrylonitrile-2-amino thiazole resin	25.9 nm	Hg, Cd, Pb, Cu, Zn, and Ni	pH	[59]
acrylic acid grafted polytetrafluoroethylene fibers	---	Er(III)	pH, temperature, initial metal ion concentration	[60]
IRN77 cation exchange resin	<0.300 mm, >1.180 mm	Co(II), Cr(III), Ni(II)	dosage, pH, stirring time, and initial concentration	[61]
polyacrylonitrile-2-amino-2-thiazole moiety resin	---	precious metal ions	temperature, pH	[62]
amines and sulfur chelating resin	---	Zn (II), Cd (II), Hg (II)	pH	[63]
polyacrylonitrile-amino thiourea resin	25.1 nm	Rh(III), Ru(IV), Pd(II), Ir(IV)	pH, functional groups	[64]
methyl glycidyl ester of acrylic resin	0.07, 0.15 and 0.06 μm	Cu(II), Pb(II)	pH	[65]
cationic polystyrene balls	---	paper anion pollutants in water	temperature, distributed control system	[68]
crosslinked polystyrene diethanolamine load DiDai type of dendritic polymers	---	metal ions	temperature, content of functional groups	[69]
grafted polytetrafluoroethylene fibers	---	Cu(II)	temperature	[70]
coal and polyvinyl chloride (PVC) scrap	150–200 μm	Hg	temperature	[78]
porous cellulose modified polyethylene imine carrier	---	Hg	the adsorption selectivity	[80]
IRN-77 cation exchange resin	<0.300 mm, >1.180 mm	Co(II), Ni(II), Cr(III)	adsorbent dosage	[89]
3-aminopyridine hydrophilic spacer chelating resin	---	Hg(II), Ag(I), Fe(III), Pb(II), Co(II), Cu(II), Ni(II), Cd(II)	the adsorption capacity	[91]
IRC748 and NDC702	36.85 and 34.53 nm	Cu(II), Pb(II), Cd(II)	pH	[100]
new IDA-chelating resin	---	Cu(II), Pb(II), Cd(II)	pH	[101]
D152 resin	10.3 nm	Cd(II)	pH, temperature	[102]
polyacrylic acid-PVC composite adsorbent	---	cadmium pollution of wastewater	pH	[103]
PS	---	Cr(VI)	aging	[104]
doped polyaniline	---	anionic dye	doping	[105]
“X” shape of the cavity 2 d coordination polymer	---	oxygen anion pollutants	the adsorption selectivity	[106]
phytic acid doped polyaniline nanofibers	80–100 nm	water-borne Cu(II)	pH	[107]
new type of sulfur-containing polyamine chelating resin	---	precious metal	The solvent, temperature, and time	[108]
electrospinning fiber membrane	---	heavy metal	porosity, specific surface area	[109]
amination polyacrylonitrile fiber	---	Pb, Cu	pH	[110]

**Table 3:** Adsorption of mixed pollutants.

Adsorbent	Surface Area	Pollutants	Influencing Factors	Reference
porous super hydrophobic foam plastic	---	oily wastewater	a small amount of span 80 and silica particles	[83]
carbonized polypropylene	---	oil	NiO catalyst diameter	[111]
weak base anion exchange resin	---	benzene sulfonate	pH	[112]
polystyrene matrix	---	protein fiber connection	the concentration of	[113]
polystyrene	---	thrombin	cone methyl sulfonate and sulfanilamide essence	[114]
La(OH)_3_@SA/PAM	---	methylene blue, crystal violet, and malachite green	ultraviolet light	[115]
metal ions impregnated polystyrene resin	---	antibiotics in water pollutants	pH	[116]
grafted polyethylene imine melamine formaldehyde	---	CO_2_	temperature	[117]
polyvinyl chloride (PVC)/polystyrene fiber electrostatic spinning	70–300 μm	oil pollution	porosity	[118]
polyvinyl chloride (PVC) mesoporous membrane	45 nm	methylene blue	porosity	[119]
2-amino modified Chloromethylated polystyrene and GQ-08 resin	9.93 and 8.99 nm	glyphosate	pH	[120]
PTFE membrane	0.1 μm	crude oil	crude oil initial concentration, contact time, pH, ionic strength, temperature	[121]
polymer nanocomposites	---	harmful pollutants in the water or wastewater	pH	[122]
low poly beta cyclodextrin coupling polystyrene	---	puerarin	solubility	[123]

## 6. Adsorption Mechanism

**Figure 5:**
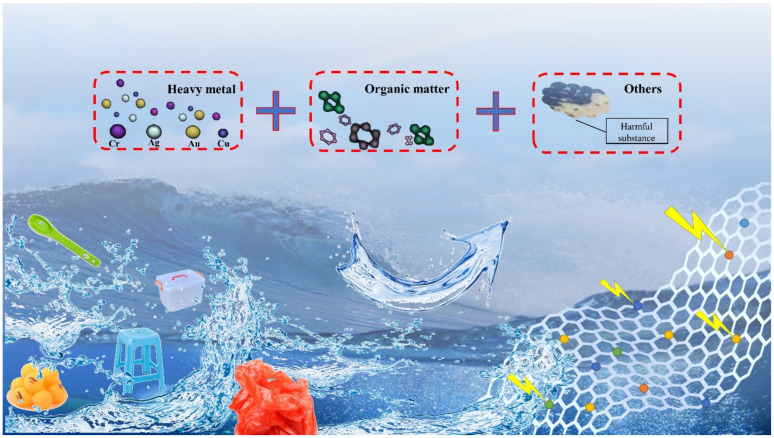
The adsorption machines in the process of adsorbing pollutants with microplastics.

Interlaced and complicated factors formed the adsorption mechanism of microplastics on pollutants. Figure 5 briefly depicts the mechanism of the pollutant adsorption process by microplastics. Based on the literature on the adsorption of plastics on various pollutants, the adsorption mechanism of plastics on pollutants include many aspects, and these are briefly summarized in Tables S3–S5: (i) The adsorption mechanism of plastic on organic pollutants as shown in Table S3; (ii) The adsorption mechanism of plastics on inorganic pollutants as shown in Table S4; and (iii) the adsorption mechanism of plastics on other pollutants as shown in Table S5. In addition, the adsorption affinity, hydrogen bonding, π–π interactions [124], and etc., as shown in Figure S2, had different results in different adsorption experiments, as follows.

### 6.1. Adsorption Affinity

The adsorption affinity of resin for pollutants was one of the main mechanisms for studying the properties of microplastics. In the adsorption process of microplastics, they often showed remarkable adsorption capacity for some contaminants. As in the process of chloromethane adsorption of plastic adsorbent, the low solubility of chloromethane was more readily adsorbed [85]. However, with the adsorption of styrene-DVB resin for homocarboxylic acids, carboxylic acids with high molecular weights were more easily adsorbed. The adsorption capacity of mercury was always higher than that of lead in the adsorption capacity of poly(ethylamine) to lead and mercury [57]. 

In the adsorption of mercury by a polyethyleneimine-modified porous cellulose carrier, the mercury amine complex was formed by a polyethyleneimine-modified porous cellulose carrier; thus, mercury had high stability, which made microplastics have a high mercury adsorption efficiency [80]. Additionally, the folate-polyaniline hybrid hydrogel can selectively adsorb anionic dyes and has a higher affinity for anionic pollutants, such as chromium (VI), eosin yellow, rose red, and methyl orange [54]. Likewise, polyacrylonitrile-2-aminothiazole resin also had a high adsorption efficiency for mercury-, cadmium-, lead-, copper-, zinc-, and nickel-polluted wastewater [59]. 

Under the condition of the coexistence of Hg(II), Ag(I), Fe(III), Pb(II), Co(II), Cu(II), Ni(II), Cd(II), and Zn(II) ions, 3-aminopyrine hydrophilic spacer arm chelating resin showed strong adsorption performance for Hg(II) [91]. Interestingly, polyvinylchloride microplastics had a higher affinity for TCEP in the adsorption of trin-butyl phosphate and tris (2-chloroethyl) phosphate [94]. When microplastics (MPs) were used to remove fungicides, the adsorption capacity of pyrrole cyclosporin was the highest, followed by microoxysporin and dioxysporin [82]. In general, polyethylene exhibited greater adsorption capacity than other types of plastics [125]. 

The role of functional groups, such as the adsorption effect of terlomycin on MPs, was in the order of PE (polyethylene) < PP (polypropylene) < PS (polystyrene) < PVC (polyvinyl chloride). In addition to its strength, the adsorption affinity sometimes needed the help of other substances. For example, silica played a vital role in treating oil-containing wastewater by porous waste plastic superhydrophobic foams, and silica had a strong affinity for both porous waste plastic superhydrophobic foams and oil-containing wastewater [125]. 

In the adsorption of iminodiacetic acid chelate resin for trivalent metal ions, Sc(III) > Ga(III) ≈ In(III) ≈ Fe(III) > Y(III) > La(III) > Al(III); however, in aqueous solution, Ga(III) > Fe(III) > In(III) > Sc(III) > Al(III) > Y(III) > La(III) [55]. In the pH range of 2–8, metal ion-impregnated polystyrene resins had a strong adsorption capacity for antibiotic contaminants in water [116]. The adsorption of Cd and Ni ions on polystyrene nanofibers in an aqueous solution can achieve the best effect [58]. Adsorption was the main mechanism of oil pollution treatment for the porous structure of electrospun polyvinyl chloride/polystyrene fiber [118].

In addition, there was adsorption selectivity, as low concentrations of polystyrene enhance the migration of nonpolar (pyrene) and weakly polar (2,2,4,4-tetrabromodiphenyl ether) compounds but had little effect on the migration of three polar compounds (Bisphenol A, bisphenol F, and 4-nonylphenol) [95]. Moreover, when Co(II), Ni(II), and Cr(III) ions coexist in a solution, Cr(III) replaced them even if Co(II) and Ni(II) were adsorbed on the resin [89].

### 6.2. Chemical Bonds

In the process of resin treatment of microplastics, the role of various chemical bonds cannot be ignored, such as hydrogen bonds, van der Waals forces, π–π interactions, phosphorus–phosphorus accumulation interactions, carbon–chlorine bonds, etc.

Hydrogen bonds were an important factor affecting the mechanism of resin adsorption. For example, the hydrogen bond formed between the electron pair on nitrogen and the hydroxyl hydrogen of molecular phenol, the hydrogen bond connected to the nitrogen of amine, and the hydrogen bond formed between the hydrogen of the hydroxyl group and the free amine group played an essential role in the adsorption performance of microplastics. The adsorption of 5-sodium disulfoisphthalic acid by acrylate polymer YWB-7 resin was a similar principle [88].

Consequently, benzene rings and other functional groups inevitably appear in resins. Under the influence of the benzene ring and functional group, polystyrene had higher adsorption performance for echinophilin [82]. In the process of the adsorption test of polyethylene microplastics to organic luminescent materials, the characteristic band of the infrared spectrum showed no change [94]. Due to these benzene ring functional groups, resin adsorption containing pollutants will produce π–π interactions. For instance, polystyrene was adsorbed to echinophorin [82]. The adsorption of phenanthrene on plastic fibers (some plastics and phenanthrene contain benzene rings) [81], plastics and microplastics, phenylene, nitrobenzene, naphthalene, and other substances played a key role in the degradation of pollutants due to the presence of benzene ring functional groups.

Moreover, when coal and waste polyvinyl chloride adsorbed mercury, the carbon–chlorine bond can be changed into chloride ions, thus, realizing the chemisorption of mercury [78]. Phosphorus–phosphorus accumulation interactions played an important role in the interaction between adsorbents and trinitrotoluene [87]. In addition, the adsorption of Cd(II) by D152 resin was also investigated. The removal efficiency of Cu(II) was significantly improved by modifying PTFE fibers by radiation grafting to make them contain C=O and -OH functional groups [70].

### 6.3. Ion Exchange

As the experiment continued, the pH remained the same; however, the pollutant was gradually absorbed, indicating that the pollutant was removed by ion exchange. As with anion exchange resins that absorb phenol [76], ion exchange between carboxyl groups of proteins and functional groups of resins in the form of acids; phenol formaldehyde anion exchange resin can not only exchange ions in the form of acid but also absorb protons in the form of the free base under the action of amine functional group of benzene sulfonate (BS) removal [112]. 

In the adsorption of phenol by ion exchange resin, alkaline conditions were the most favorable [53]. Ion exchange of charged carboxyl groups of polyacrylic acid with cadmium(II) solution [103]. The iminodiacetic acid chelating resin had an ion exchange effect in the adsorption of Cu(II), Pb(II), and Cd(II) ions [101].

### 6.4. Hydrophobic Interaction

As hydrophobic organic matter, microplastics had abnormal adsorption capacity for hydrophobic organic matter [126], hydrophobic substances are more easily removed from aqueous solutions than hydrophilic substances [85]. For example, polythioctanoid-based coagulants preferred compounds with positive octanol-water partitioning coefficients [55]. The adsorption capacity of ectocystine on polyethylene was in the order of pyrazolesterim, dixoxystim, and pyrazolesterim. Between the functionalized polystyrene nanospheres and 2,4,6-trinitrotoluene, the adsorption capacity of polystyrene and KH570-polystyrene nanospheres for trinitrotoluene was the highest [87]. 

In the adsorption experiment of chlorinated methane by plastic, GAC > PLJ-PVC > NEO > PE [85]. Perfluorinated octane sulfonic acid (FOSA) was more easily adsorbed on polyethylene [52]. In the adsorption of three triazole fungicides by polystyrene microplastics, the resin with strong hydrophobicity had more substantial adsorption power [50]. Benzene derivatives were more hydrophobic than benzene monomers, therefore they were polyethylene rather than other polymer resins with higher adsorption properties [49]. 

In the adsorption of porous polycalixarene to organic micropollutants, CalP4 showed ultrafine adsorption [79]. After the adsorption of phenanthrene and its mono hydroxyl derivatives, the bands of PVC microplastics did not change significantly [90]. The adsorption efficiency of 5-sodium disulfoisylphthalic acid by acrylate polymer YWB-7 resin was higher in an aqueous solution without methanol [88].

### 6.5. Role of the Environment

In addition to the adsorption of pollutants by microplastics themselves, aging and other factors can enhance the adsorption performance of microplastics [127]. The adsorption effect of Cu(II) on metal ions was the best in the experiments of cross-linked polystyrene-supported low-generation diethanolamine dendrimers [69]. IDA-chelating resin had the best adsorption effect on Cu(II) and the worst adsorption effect on Cd(II) [100]. There were many kinds of microplastics, such as polyamide, rubber, and glass, and similar to the adsorption of metal pollutants, the adsorption effect was the best for polyamide and the worst for glass [30]. 

In the adsorption of Co(II), Cr(III), and Ni(II) by IRN77 cation exchange resin, Cr(III) was mainly adsorbed. Even though IRN77 cation exchange resin will adsorb Co(II) and Ni(II) at the beginning, it will desorb after some time, and the adsorption site is replaced by Cr(III) [61]. Among the adsorptions of rhodium(III), ruthenium(IV), iridium(IV), and palladium(II), the adsorption of polyacrylonitrile-ATAL was the highest for rhodium(III) [64]. Polyacrylonitrile-thiosemicarbazone resin had excellent adsorption selectivity for Rh(III), Ru(IV), Pd(II), and Ir(IV) [64]. In the adsorption of Cu(II) and Pb(II) by amine chelating resin, the absorption effect of copper was higher than that of lead under any conditions [63].

### 6.6. Electrostatic Interaction

Electrostatic interactions were due to the process of plastic adsorption, adsorption, and pollutants due to attraction or repelling of each other with charge. Glass-reinforced plastics, for example, were positively charged in acidic conditions and had a strong electrostatic attraction to acrylic polyamphoteric electrolytes [77]. At low pH, primary amines, secondary amines, and imines in folate-polyaniline hybrid hydrogels had groups containing holes, which can be protonated to form positively charged cations (e.g., –NH_3_^+^, –NH_2_^+^, –NH^+^, etc.), these cationic groups had an electrostatic attraction with Cr(VI), Hg(II), and Pb(II), thus, improving the removal efficiency [54]. 

The positively charged cetyltrimethylammonium bromide can effectively adsorb anions in the presence of surfactants [84]. The adsorption of perfluorooctanesulfonamides by polyethylene microplastics was not affected by ionic strength; however, the influence of ionic strength was evident after ionization of pollutants, which was the effect of electrostatic force. Due to the pH value, the electrostatic interaction with pollutants affected the adsorption of three triazole fungicides by polystyrene microplastics [50].

### 6.7. Other Adsorption Mechanisms

In addition to the above common mechanisms of pollutant adsorption of several microplastics, there were also carboxyl functional groups in acrylic grafted PTFE fibers, which can bind with Er(III). Metal ions Er(III) have empty orbitals, and carboxyl groups provide their electron pairs, which belong to complexation. Under the action of surface-initiated free radicals, cationic substances can be connected on the surface of polystyrene spheres to obtain cationic microplastics and can then act as high-efficiency adsorption anionic pollutants in white papermaking water, which was grafted [68]. The properties of chelating groups and metal-ligand CO stability, such as amine and sulfur chelating resin for Cu(II) and Pb(II) adsorption [63]. The chelating mechanism of polyethylamine with lead and mercury are all examples of this [57].

## 7. Conclusions

### 7.1. Summary

The reasons and mechanisms of plastic adsorption for organic, inorganic, and mixed pollutants were discussed. There are many reasons affecting sewage treatment in using plastic. The influence of plastics includes many factors. As far as the most typical pH was concerned, it was divided into three aspects: (i) The existence of electrostatic repulsion at low pH reduced the adsorption efficiency. (ii) The adsorption efficiency was the highest when the pH was between 3 and 6. (iii) Precipitation at a high pH value. However, there were certain exceptions as mentioned earlier. In addition, the actions of the environment, illumination, weathering, crushing, etc. further increase the contact area between microplastics and pollutants.

The mechanism of sewage treatment by using microplastics was also complicated and interrelated. We found that the main mechanism of sewage treatment by using microplastics was mainly divided into: (i) Plastics naturally had other properties, such as showing a cationic effect, high-efficiency adsorption, and a porous structure, and they showed a unique and strong affinity for pollutants; (ii) microplastics with carboxylic acid, amino groups, and other functional groups had hydrogen bonds and electrostatic attraction with pollutants; and (iii) the pore-carbon structure benzene ring of microplastics could produce π–π interactions or phosphorus–phosphorus stacking interactions.

At present, microplastics have great potential in sewage treatment. In addition to the adsorption capacity (hydrophobic), adsorption efficiency, and other advantages, microplastics in sewage treatment have reusable benefits in comparison to other adsorbents. The excellent performance of microplastics in sewage treatment is still being investigated. In addition to sewage treatment, microplastics have been applied in industry. Microplastics will be even more valuable in the future.

### 7.2. Prospect

Adsorption technology is gradually applied to all aspects of environmental treatment, and it occupies a dominant position in sewage treatment. In this paper, the adsorption mechanism and influencing factors of different adsorbents for different pollutants were summarized as well as the activities of microplastics in soil and water environments. These have reference value for the study of microplastic adsorption. Although the influence of the water environment, soil fractionation, and speciation on the microplastic adsorption process are not completely clear, we hope that large-scale, sustainable, and low-cost adsorption technologies will be developed.

## Data Availability

Not applicable.

## Supplementary Materials

The following supporting information can be downloaded at: https://www.mdpi.com/article/10.3390/nano12132256/s1, Figure S1: The process of forming large plastics into microplastics, Figure S2: Schematic representation of the six sorption mechanisms, Table S1: Classification of microplastics, Table S2: Surface area parameters of different adsorbents, Table S3: Adsorption mechanism of organic pollutants, Table S4: Adsorption mechanism of inorganic pollutants, and Table S5: Adsorption mechanism of other pollutants [128,129,130,131,132,133,134].
