# Supplementary material for: Adsorption of Different Pollutants by Using Microplastic with Different Influencing Factors and Mechanisms in Wastewater: A Review
Source: Nanomaterials (Basel). 2022 Jun 30;12(13):2256. doi: 10.3390/nano12132256 (PMC9268391; doi:10.3390/nano12132256)
Supplement: Supplementary file 1 [file nanomaterials-12-02256-s001.zip › nanomaterials-1780412-supplementary.pdf]

Supplementary Material

Figure S1. The process of forming large plastics into microplastics **[1]**.

Figure S2. Schematic representation of the six sorption mechanisms **[2]**.

Table S1. Classification of microplastics.

Table S2. Surface area parameters of different adsorbents.

Table S3. Adsorption mechanism of plastic on organic pollutants.

Table S4. Adsorption mechanism of plastics on inorganic pollutants.

Table S5. Adsorption mechanism of plastics on other pollutants.

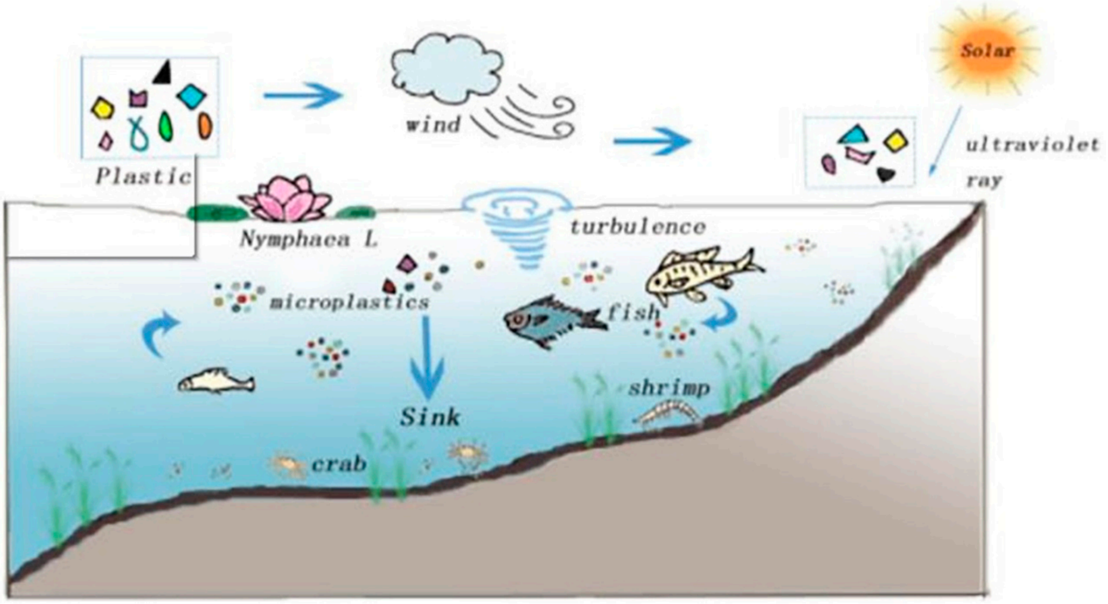

Figure S1. The process of forming large plastics into microplastics **[1].**

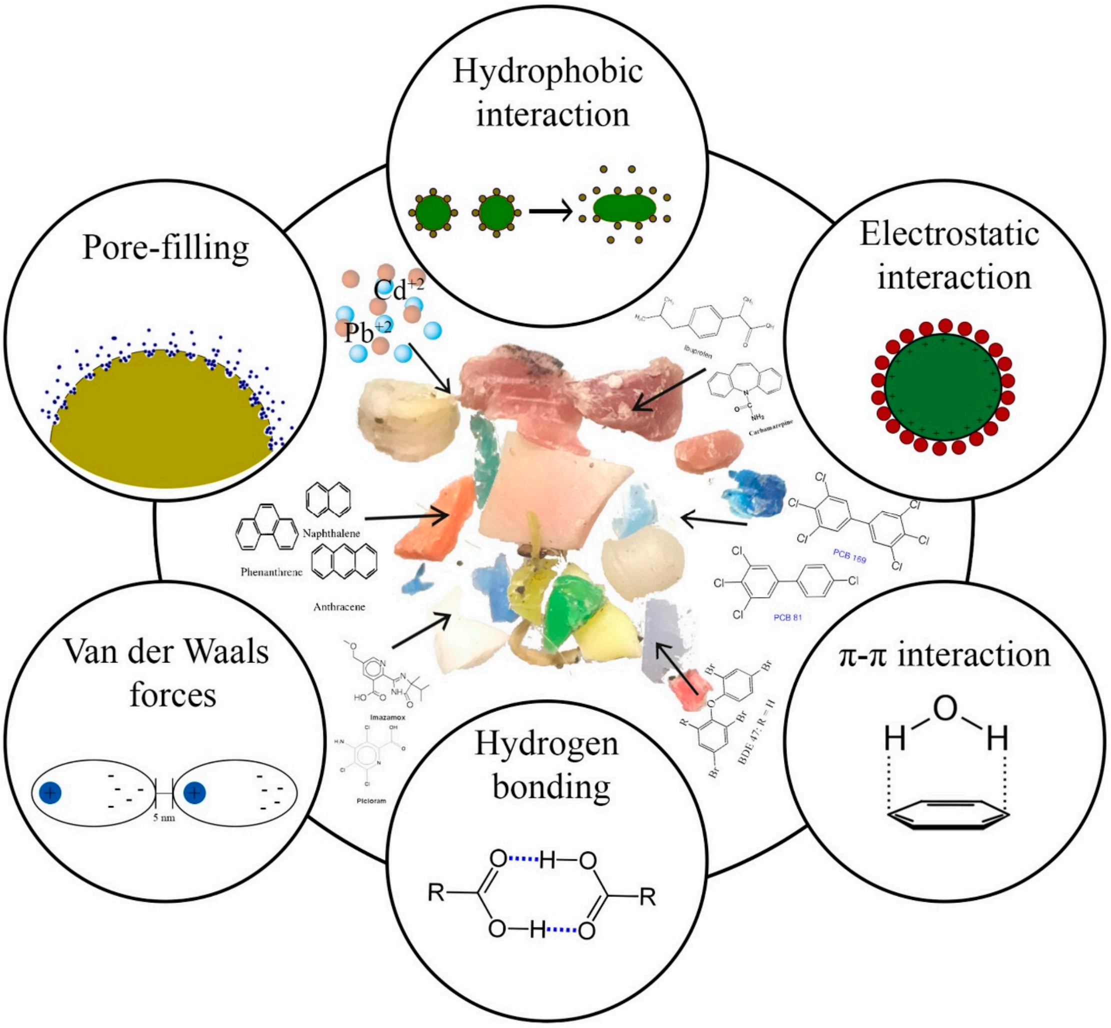

Figure S2. Schematic representation of the six sorption mechanisms **[2].**

Table S1. Classification of microplastics.

Features	Classification	Reference
source	primary plastic (primary plastic), secondary plastic	**[3]**
color	white/translucent, black/brown, blue, red/pink, green	**[3]**
Shape	fibers, filaments, fragments, films, particles	**[4]**
polymer	polyethylene, polyethylene terephthalate, polyester, polypropylene plastic polymer	**[5]**
Source (grade)	foam plastics (polycarbonate polyurethane (PCU) fragments), boat paints (polypropylene PP, polyethylene PE + film PP), fishing nets and lines, and textile production (PET, PES)	**[6]**
particle size	<0.5 mm, 0.5 mm - 1 mm, 1 mm - 2 mm, 2 mm - 3 mm, 3 mm - 4 mm, 4 mm - 5 mm	**[7]**

Table S2. Surface area parameters of different adsorbents

Adsorbent	surface area/ m^2/g	Reference
HDPE and LDPE	0.164 ± 0.02 and 0.043 ± 0.001	**[8]**
MN 200, Dowex XZ and AuRIX	793, 39.2 and 37.5	**[9]**
FRP particles	2.7	**[10]**
CalP2, CalP3 and CalP4	596, 630 and 759	**[11]**
PE rope of 1, 4 and 10-mm	0.129, 0.042 and 0.027	**[12]**
YWB-7	663.8	**[13]**
PVC	0.595	**[14]**
PE, PP, PES and PA	0.2651 ± 0.01, 0.3553 ± 0.04, 0.1127 ± 0.03 and 0.1170 ± 0.03	**[15]**
Synthetic Resin	27.82	**[16]**
PAN	27.80	**[17]**
PAN	27.80	**[18]**
Resin	47.54, 39.15 and 191.57	**[19]**
IRC748 and NDC702	36.3985 and 14.2367	**[20]**
D152 resin	4.8	**[21]**
Cl-PAni and Ph-PAni NFs	37.6 and 44.1	**[22]**
Electrospun fibrous	10-40	**[23]**
PE, PET, PP, PS and PVC	1.4, 0.35, 2.7, 3.2 and 0.59	**[24]**
grafted polyethylene imine melamine formaldehyde	460	**[25]**
Chloromethylated polystyrene and GQ-08 resin	24.76 and 31.45	**[26]**

Table S3. Adsorption mechanism of organic pollutants.

Adsorbent	Pollutants	The adsorption mechanism	Reference
polyethylene microplastic	aromatic amine	the salting out effect	**[8]**
polymer resin	phenol	the mechanism of adsorption and ion exchange	**[9]**
glass fiber reinforced plastic	polyacrylic acid	electrostatic interaction	**[10]**
porous polycarboxarene	organic micropollutants	hydrophobic interactions	**[11]**
microplastic	phenanthrene	absorption mechanism, the hydrophobicity of organic carbon	**[12]**
acrylate polymer YWB - 7 resin	5 - 2 sulfo sodium isophthalic acid	hydrogen bonding and hydrophobic interaction	**[13]**
micro polyvinyl chloride (PVC) plastic	Fe and single hydroxyl derivatives	hydrophobic interactions	**[14]**
biological membrane reinforced plastic microfiber	perfluorinated octane sulfonic acid (PFOS)	hydrophobic interactions	**[15]**
Micro polystyrene plastics	three three azole fungicide	hydrophobic and electrostatic interactions	**[27]**
plastic cellulose acetate	2, 4 - diallyl (sorbic acid)	group acetylated	**[28]**
microplastic	PFOS, FOSA	hydrophobic interactions	**[29]**
polylipoic acid ester-base coagulant	organic pollutants (pops)	hydrophobic interactions	**[30]**
micro polystyrene plastics	soluble organic matter (DOM)	hydrophobic interactions, π-π electron donor receptor interactions, salting-out effect	**[31]**
micro polystyrene plastics	triclosan	hydrophobic interactions	**[32]**
polyethylene and polyvinyl	sanzheng butyl ester phosphate and phosphate (2-	weak van der Waals force, adsorption selectivity	**[33]**

chloride (PVC) plastic	ethyl chloride)		
in plastic and plastic	the philippines, nitrobenzene and naphthalene	hydrophobic interaction, π-π, crystallinity	**[34]**
anion exchange resin	phenol	ion exchange	**[35]**
microplastic PS + PE	three types of cones sterol fungicide	hydrophobic interaction, van der Waals force	**[36]**
the original rusty water microplastic	ions, organic pollutants	electrostatic force, van der Waals force, space steric hindrance	**[37]**
functionalized polystyrene nano ball	2,4,6-trinitrotoluene (TNT)	p - phosphorus accumulation interaction, hydrogen, and hydrophobic interaction	**[38]**
polystyrene	organic pollutants (pops)	the adsorption affinity	**[39]**
plastic adsorbent	chlorinated methane	the adsorption selectivity	**[40]**
polystyrene microspheres	naphthalene (NAP) and its derivatives	hydrophobic	**[41]**
microplastic	17 beta estradiol	hydrophobic	**[42]**
microplastic	nonpolar organic compounds	hydrophobic interactions	**[43]**
microplastic	fuel aromatics and ether	hydrophobic	**[44]**
microplastic	phenanthrene	hydrophobic	**[12]**
polystyrene	nonionic organic compounds	π-π- aromatic interactions, hydrophobic interactions	**[45]**

Table S4. Adsorption mechanism of inorganic pollutants.

Adsorbent	Pollutants	The adsorption mechanism	Referenc e
polyacrylonitrile - 2 - amino thiazole resin	Hg, Cd, Pb, Cu, Zn and Ni	the adsorption affinity	**[16]**
polyacrylonitrile - 2 - amino - 2 - thiazole moiety resin	precious metal ions	the adsorption affinity	**[17]**
polyacrylonitrile - amino thiourea resin	Rh(III), Ru(IV), Pd(II), Ir(IV)	the adsorption affinity	**[18]**
methyl glycidyl ester of acrylic resin	Cu(II), Pb(II)	the adsorption affinity	**[19]**
IDA - chelating resin	Cu(II), Pb(II), Cd(II)	the adsorption affinity	**[20]**
D152 resin	Cd(II)	surface adsorption, surface electrical property, and hydrogen bond	**[21]**
phytic acid doped polyaniline nanofibers	water-borne Cu (II)	the adsorption affinity	**[22]**
electrospinning fiber membrane	heavy metal	the adsorption affinity	**[23]**
microplastic	Cd, Co, Cr, Cu, Ni, Pb, and Zn	chemical adsorption	**[24]**
iminodiacetic acid chelating resin	Sc(III), Y(III), La(III), Fe(III), Al(III), Ga(III), In(III)	the adsorption selectivity	**[30]**
micro/nano plastic	metal	the adsorption affinity	**[32]**
folic acid - polyaniline hybrid hydrogel	Cr (VI), Hg (II), Pb (II)	REDOX	**[46]**
folic acid - polyaniline hybrid hydrogel	anionic dye	the adsorption affinity	**[46]**
modification of polyethyleneimine	Cr(III), Fe(III)	chemical adsorption	**[47]**
polyethylimine	Pb and Hg	the adsorption affinity	**[48]**

polystyrene nanofibers	Cd, Ni	normal adsorption	**[49]**
acrylic acid grafted polytetrafluoroethylene fibers	Er(III)	ion exchange resin and metal or complex	**[50]**
new IDA - chelating resin	Cu(II), Pb(II), Cd(II)	ion exchange, chemical interaction	**[51]**
IRN77 cation exchange resin	Co(II), Cr(III), Ni(II)	the adsorption affinity	**[52]**
amines and sulfur chelating resin	Zn (II), Cd (II), Hg (II)	the nature of the chelating groups, the stability of the metal-ligand CO	**[53]**
coal and polyvinyl chloride (PVC) scrap	Hg	carbon chlorine key (modified)	**[54]**
cationic polystyrene balls	paper anion pollutants in water	grafting	**[55]**
crosslinked polystyrene diethanolamine load DiDai type of dendritic polymers	metal ions	hydrogen bonding, adsorption affinity	**[56]**
grafted polytetrafluoroethylene fibers	Cu(II)	the hydroxyl and carbonyl	**[57]**
porous cellulose modified polyethylene imine carrier	Hg	the adsorption affinity	**[58]**
IRN - 77 cation exchange resin	Co(II), Ni(II), Cr(III)	the adsorption affinity	**[59]**
3 - aminopyridine hydrophilic spacer chelating resin	Hg(II), Ag(I), Fe(III), Pb(II), Co(II), Cu(II), Ni(II), Cd(II)	the adsorption selectivity	**[60]**
polystyrene (MPS)	nanometer oxide (CeNPs)	the adsorption affinity	**[61]**
polyacrylic acid - PVC composite adsorbent	cadmium pollution of wastewater	ion exchange	**[62]**
micro polystyrene plastics	heavy metal	physical and chemical adsorption	**[63]**
microplastic	metal pollutants	the adsorption	**[64]**
doped polyaniline	anionic dye	chemical interaction	**[65]**
"X" shape of the cavity 2 d coordination polymer	oxygen anion pollutants	the adsorption affinity	**[66]**
new type of sulfur-containing polyamine chelating resin	precious metal	the adsorption affinity	**[67]**

amination polyacrylonitrile fiber	Pb, Cu	the adsorption affinity	**[68]**
micro polystyrene plastics	As(III)	hydrogen bonding	**[69]**

Table S5. Adsorption mechanism of other pollutants.

Adsorbent	Pollutants	The adsorption mechanism	Reference
grafted polyethylene imine melamine formaldehyde	CO_2	chemical adsorption and physical adsorption	**[25]**
2-amino modified polystyrene pyridine resin, D301 resin, and 330 resin	glyphosate	hydrogen bonding, single anion exchange	**[26]**
porous super hydrophobic foam plastic	oily wastewater	Superhydrophobic and superhydrophilic	**[70]**
carbonized polypropylene	oil	mechanism of carbide	**[71]**
weak base anion exchange resin	benzene sulfonate	ion exchange	**[72]**
polystyrene matrix	protein fiber connection	hydrophobic interactions	**[73]**
polystyrene	thrombin	the adsorption affinity	**[74]**
$La(OH)_3$@SA/PAM	methylene blue, crystal violet, and malachite green	hydrogen bonding, electrostatic interaction	**[75]**
metal ions impregnated polystyrene resin	antibiotics in water pollutants	the surface complexation, cationic bridge interaction, and electrostatic attraction/competition	**[76]**

polyvinyl chloride (PVC)/polystyrene fiber electrostatic spinning	oil pollution	adsorption and capillary action	**[77]**
polyvinyl chloride (PVC) mesoporous membrane	methylene blue	high water flux	**[78]**
micro polystyrene plastics	ator-vastatin (ATV) and amlodipine (AML)	hydrophobic interaction, π-π	**[79]**
PTFE membrane	crude oil	physical adsorption	**[80]**
polymer nanocomposites	harmful pollutants in the water or wastewater	the adsorption affinity	**[81]**
low poly beta cyclodextrin coupling polystyrene	puerarin	hydrogen bonding, hydrophobic effect, van der Waals force, electrostatic and π-π synergy of interaction	**[82]**

References

1. Tang, Y.; Liu, Y.; Chen, Y.; Zhang, W.; Zhao, J.; He, S.; Yang, C.; Zhang, T.; Tang, C.; Zhang, C., A review: Research progress on microplastic pollutants in aquatic environments. *Sci. Total Environ.* **2021,** *766*, 142572 https://doi.org/10.1016/j.scitotenv.2020.142572
2. Torres, F. G.; Dioses-Salinas, D. C.; Pizarro-Ortega, C. I.; De-la-Torre, G. E., Sorption of chemical contaminants on degradable and non-degradable microplastics: Recent progress and research trends. *Sci. Total Environ.* **2021,** *757*, 143875 https://doi.org/10.1016/j.scitotenv.2020.143875
3. Migwi, F. K.; Ogunah, J. A.; Kiratu, J. M., Occurrence and spatial distribution of microplastics in the surface waters of Lake Naivasha, Kenya. *Environ. Toxicol. Chem.* **2020,** *39*, 765-774 https://doi.org/10.1002/etc.4677
4. Yang, Z.; Li, S.; Ma, S.; Liu, P.; Peng, D.; Ouyang, Z.; Guo, X., Characteristics and removal efficiency of microplastics in sewage treatment plant of Xi🅐an City, northwest China. *Sci. Total Environ.* **2021,** *771*, 145377 https://doi.org/10.1016/j.scitotenv.2021.145377
5. Qin, Y.; Wang, Z.; Li, W.; Chang, X.; Yang, J.; Yang, F., Microplastics in the sediment of lake Ulansuhai of Yellow river basin, China. *Water Environ. Res.* **2020,** *92*, 829-839 https://doi.org/10.1002/wer.1275
6. Frias, J.; Sobral, P.; Ferreira, A. M., Organic pollutants in microplastics from two beaches of the Portuguese coast. *Mar. Pollut. Bull.* **2010,** *60,* 1988-1992 https://doi.org/10.1016/j.marpolbul.2010.07.030
7. Cole, M.; Lindeque, P.; Halsband, C.; Galloway, T. S., Microplastics as contaminants in the marine environment: a review. *Mar. Pollut. Bull.* **2011,** *62,* 2588-2597 https://doi.org/10.1016/j.marpolbul.2011.09.025
8. Vockenberg, T.; Wichard, T.; Ueberschaar, N.; Franke, M.; Stelter, M.; Braeutigam, P., The sorption behaviour of amine micropollutants on polyethylene microplastics–impact of aging and interactions with green seaweed. *Environ. Sci-Proc Imp* **2020,** *22*, 1678-1687 https://doi.org/10.1039/D0EM00119H
9. Caetano, M.; Valderrama, C.; Farran, A.; Cortina, J. L., Phenol removal from aqueous solution by adsorption and ion exchange mechanisms onto polymeric resins. *J. Colloid Interface Sci.* **2009,** *338,* 402-409 https://doi.org/10.1016/j.jcis.2009.06.062

10. Terabayashi, T.; Maruyama, T.; Shimizu, Y.; Komatsu, M.; Takahashi, T., Surface modification with acrylic polyampholytes 1, adsorption of acrylic polyampholytes on fiberglass reinforced plastics, and characterization of the polymer adsorbed surfaces. *J. Appl. Polym. Sci.* **2006,** *101*, 4454-4461 https://doi.org/10.1002/app.24084
11. Shetty, D.; Jahovic, I.; Raya, J.; Asfari, Z.; Olsen, J.-C.; Trabolsi, A., Porous polycalix [4] arenes for fast and efficient removal of organic micropollutants from water. *ACS Appl. Mater. Interfaces* **2018,** *10*, 2976-2981 https://doi.org/10.1021/acsami.7b16546
12. Wang, Z.; Chen, M.; Zhang, L.; Wang, K.; Yu, X.; Zheng, Z.; Zheng, R., Sorption behaviors of phenanthrene on the microplastics identified in a mariculture farm in Xiangshan Bay, southeastern China. *Sci. Total Environ.* **2018,** *628*, 1617-1626 https://doi.org/10.1016/j.scitotenv.2018.02.146
13. Yang, W.; Li, A.; Zhang, Q.; Fei, Z.; Liu, F., Adsorption of 5-sodiosulfoisophthalic acids from aqueous solutions onto acrylic ester polymer YWB-7 resin. *Sep. Purif. Technol.* **2005,** *46*, 161-167 https://doi.org/10.1016/j.seppur.2005.05.005
14. Bao, Z.-Z.; Chen, Z.-F.; Zhong, Y.; Wang, G.; Qi, Z.; Cai, Z., Adsorption of phenanthrene and its monohydroxy derivatives on polyvinyl chloride microplastics in aqueous solution: Model fitting and mechanism analysis. *Sci. Total Environ.* **2021,** *764*, 142889 https://doi.org/10.1016/j.scitotenv.2020.142889
15. Bhagwat, G.; Tran, T. K. A.; Lamb, D.; Senathirajah, K.; Grainge, I.; O'Connor, W.; Juhasz, A.; Palanisami, T., Biofilms enhance the adsorption of toxic contaminants on plastic microfibers under environmentally relevant conditions. *Environ. Sci. Technol.* **2021,** *55*, 8877-8887 https://doi.org/10.1021/acs.est.1c02012
16. Xiong, C.; Jia, Q.; Chen, X.; Wang, G.; Yao, C., Optimization of polyacrylonitrile-2-aminothiazole resin synthesis, characterization, and its adsorption performance and mechanism for removal of Hg(II) from aqueous solutions. *Ind. Eng. Chem. Res.* **2013,** *52*, 4978-4986 https://doi.org/10.1021/ie3033312
17. Chen, Y.; Zhao, Y., Synthesis and characterization of polyacrylonitrile-2-amino-2-thiazoline resin and its sorption behaviors for noble metal ions. *React. Funct. Polym.* **2003,** *55*, 89-98 https://doi.org/10.1016/S1381-5148(02)00218-3
18. Chen, Y.-Y.; Liang, C.; Chao, Y., Synthesis and characterization of polyacrylonitrile-thiosemicarbazide resin and its sorption behavior for Rh(III) Ru(IV) Pd(II) and Ir(IV) ions. *React. Funct. Polym.* **1998,** *36*, 51-58 https://doi.org/10.1016/S1381-5148(97)00111-9
19. Atia, A. A.; Donia, A. M.; Abou-El-Enein, S. A.; Yousif, A. M., Studies on uptake behaviour of copper(II) and lead(II) by amine chelating resins with different textural properties. *Sep. Purif. Technol.* **2003,** *33*, 295-301 https://doi.org/10.1016/S1383-5866(03)00089-3
20. Ling, P.; Liu, F.; Li, L.; Jing, X.; Yin, B.; Chen, K.; Li, A., Adsorption of divalent heavy metal ions onto IDA-chelating resins: simulation of physicochemical structures and elucidation of interaction mechanisms. *Talanta* **2010,** *81*, 424-432 https://doi.org/10.1016/j.talanta.2009.12.019
21. Xiong, C.; Yao, C., Study on the adsorption of cadmium(II) from aqueous solution by D152 resin. *J. Hazard. Mater.* **2009,** *166*, 815-820 https://doi.org/10.1016/j.jhazmat.2008.11.082
22. Kim, H. J.; Im, S.; Kim, J. C.; Hong, W. G.; Shin, K.; Jeong, H. Y.; Hong, Y. J., Phytic acid doped polyaniline nanofibers for enhanced aqueous copper(II) adsorption capability. *ACS Sustain. Chem. Eng.* **2017,** *5*, 6654-6664 https://doi.org/10.1021/acssuschemeng.7b00898
23. Huang, Y.; Miao, Y. E.; Liu, T., Electrospun fibrous membranes for efficient heavy metal removal. *J. Appl. Polym. Sci.* **2014,** *131*, https://doi.org/10.1002/app.40864
24. Godoy, V.; Blázquez, G.; Calero, M.; Quesada, L.; Martín-Lara, M., The potential of microplastics as carriers of metals. *Environ. Pollut.* **2019,** *255*, 113363 https://doi.org/10.1016/j.envpol.2019.113363
25. Yin, F.; Peng, P.; Mo, W.; Chen, S.; Xu, T., The preparation of a porous melamine–formaldehyde adsorbent grafted with polyethyleneimine and its CO_2 adsorption behavior. *New J. Chem.* **2017,** *41*, 5297-5304 https://doi.org/10.1039/C7NJ00240H
26. Xiao, G.; Wen, R., Comparative adsorption of glyphosate from aqueous solution by 2-aminopyridine modified polystyrene resin, D301 resin and 330 resin: influencing factors, salinity resistance and mechanism. *Fluid Phase Equilibria* **2016,** *411*, 1-6 https://doi.org/10.1016/j.fluid.2015.11.026
27. Fang, S.; Yu, W.; Li, C.; Liu, Y.; Qiu, J.; Kong, F., Adsorption behavior of three triazole fungicides on polystyrene microplastics. *Sci. Total Environ.* **2019,** *691*, 1119-1126 https://doi.org/10.1016/j.scitotenv.2019.07.176
28. Saski, W., Adsorption of sorbic acid by plastic cellulose acetates. *J. Pharm. Sci.* **1963,** *52*, 264-268 https://doi.org/10.1002/jps.2600520316
29. Wang, F.; Shih, K. M.; Li, X. Y., The partition behavior of perfluorooctanesulfonate (PFOS) and perfluorooctanesulfonamide (FOSA) on microplastics. *Chemosphere* **2015,** *119*, 841-847 https://doi.org/10.1016/j.chemosphere.2014.08.047

30. Yuchi, A.; Sato, T.; Morimoto, Y.; Mizuno, H.; Wada, H., Adsorption mechanism of trivalent metal ions on chelating resins containing iminodiacetic acid groups with reference to selectivity. *Anal. Chem.* **1997,** *69*, 2941-2944 https://doi.org/10.1021/ac9612685
31. Abdurahman, A.; Cui, K.; Wu, J.; Li, S.; Gao, R.; Dai, J.; Liang, W.; Zeng, F., Adsorption of dissolved organic matter (DOM) on polystyrene microplastics in aquatic environments: kinetic, isotherm and site energy distribution analysis. *Ecotoxicol. Environ. Saf.* **2020,** *198,* 110658 https://doi.org/10.1016/j.ecoenv.2020.110658
32. Yu, F.; Yang, C.; Zhu, Z.; Bai, X.; Ma, J., Adsorption behavior of organic pollutants and metals on micro/nanoplastics in the aquatic environment. *Sci. Total Environ.* **2019,** *694,* 133643 https://doi.org/10.1016/j.scitotenv.2019.133643
33. Chen, S.; Tan, Z.; Qi, Y.; Ouyang, C., Sorption of tri-n-butyl phosphate and tris (2-chloroethyl) phosphate on polyethylene and polyvinyl chloride microplastics in seawater. *Mar. Pollut. Bull.* **2019,** *149,* 110490 https://doi.org/10.1016/j.marpolbul.2019.110490
34. Wang, J.; Liu, X.; Liu, G., Sorption behaviors of phenanthrene, nitrobenzene, and naphthalene on mesoplastics and microplastics. *Environ. Sci. Pollut. Res. Int.* **2019,** *26,* 12563-12573 https://doi.org/10.1007/s11356-019-04735-9
35. Pollio, F. X.; Kunin, R., Sorption of phenols by anion-exchange resin. *Environ. Sci. Technol.* **1967,** *1,* 160-163 https://doi.org/10.1021/es60002a006
36. Hai, N.; Liu, X.; Li, Y.; Kong, F.; Fang, S., Effects of Microplastics on the Adsorption and Bioavailability of Three Strobilurin Fungicides. *ACS Omega* **2020,** *5,* 30679-30686 https://doi.org/10.1021/acsomega.0c04787
37. Xia, Y.; Zhou, J.-J.; Gong, Y.-Y.; Li, Z.-J.; Zeng, E. Y., Strong influence of surfactants on virgin hydrophobic microplastics adsorbing ionic organic pollutants. *Environ. Pollut.* **2020,** *265,* 115061 https://doi.org/10.1016/j.envpol.2020.115061
38. Zhang, Y.; Wang, X.; Lv, F.; Chu, P. K.; Ye, Z.; Zhou, F.; Zhang, R.; Wei, F., Adsorption behavior and mechanism of 2, 4, 6-trinitrotoluene by functionalized polystyrene nanospheres. *J. Appl. Polym. Sci.* **2013,** *128,* 3720-3725 https://doi.org/10.1002/app.38424
39. Liu, J.; Ma, Y.; Zhu, D.; Xia, T.; Qi, Y.; Yao, Y.; Guo, X.; Ji, R.; Chen, W., Polystyrene nanoplastics-enhanced contaminant transport: role of irreversible adsorption in glassy polymeric domain. *Environ. Sci. Technol.* **2018,** *52,* 2677-2685 https://doi.org/10.1021/acs.est.7b05211
40. Cook Jr, F. C.; Hartz, K. E., Adsorption of chlorinated methanes from aqueous solution on selected plastic adsorbents. *J. - Am. Water Works Assn.* **1983,** *75,* 423-426 https://doi.org/10.1002/j.1551-8833.1983.tb05180.x
41. Yu, H.; Yang, B.; Waigi, M. G.; Peng, F.; Hu, X., The effects of functional groups on the sorption of naphthalene on microplastics. *Chemosphere* **2020,** *261,* 127592 https://doi.org/10.1016/j.chemosphere.2020.127592
42. Li, H.; Xiong, J.; Xiao, T.; Long, J.; Wang, Q.; Li, K.; Liu, X.; Zhang, G.; Zhang, H., Biochar derived from watermelon rinds as regenerable adsorbent for efficient removal of thallium(I) from wastewater. *Process Saf. Environ. Prot.* **2019,** *127,* 257-266 https://doi.org/10.1016/j.psep.2019.04.031
43. Hüffer, T.; Hofmann, T., Sorption of non-polar organic compounds by micro-sized plastic particles in aqueous solution. *Environ. Pollut.* **2016,** *214,* 194-201 https://doi.org/10.1016/j.envpol.2016.04.018
44. Müller, A.; Becker, R.; Dorgerloh, U.; Simon, F.-G.; Braun, U., The effect of polymer aging on the uptake of fuel aromatics and ethers by microplastics. *Environ. Pollut.* **2018,** *240,* 639-646 https://doi.org/10.1016/j.envpol.2018.04.127
45. Uber, T. H.; Hüffer, T.; Planitz, S.; Schmidt, T. C., Sorption of non-ionic organic compounds by polystyrene in water. *Sci. Total Environ.* **2019,** *682,* 348-355 https://doi.org/10.1016/j.scitotenv.2019.05.040
46. Das, S.; Chakraborty, P.; Ghosh, R.; Paul, S.; Mondal, S.; Panja, A.; Nandi, A. K., Folic acid-polyaniline hybrid hydrogel for adsorption/reduction of chromium(VI) and selective adsorption of anionic dye from water. *ACS Sustain. Chem. Eng.* **2017,** *5,* 9325-9337 https://doi.org/10.1021/acssuschemeng.7b02342
47. He, Z.; Song, H.; Cui, Y.; Zhu, W.; Du, K.; Yao, S., Porous spherical cellulose carrier modified with polyethyleneimine and its adsorption for Cr (III) and Fe(III) from aqueous solutions. *Chin. J. Chem. Eng.* **2014,** *22,* 984-990 https://doi.org/10.1016/j.cjche.2014.07.001
48. Delacour, M. L.; Gailliez, E.; Bacquet, M.; Morcellet, M., Poly (ethylenimine) coated onto silica gels: adsorption capacity toward lead and mercury. *J. Appl. Polym. Sci.* **1999,** *73,* 899-906 https://doi.org/10.1002/(SICI)1097-4628(19990808)73:6<899::AID-APP6>3.0.CO;2-O
49. Bahramzadeh, A.; Zahedi, P.; Abdouss, M., Acrylamide-plasma treated electrospun polystyrene nanofibrous adsorbents for cadmium and nickel ions removal from aqueous solutions. *J. Appl. Polym. Sci.* **2016,** *133,* https://doi.org/10.1002/app.42944

50. Xiong, C.; Yao, C., Preparation and application of acrylic acid grafted polytetrafluoroethylene fiber as a weak acid cation exchanger for adsorption of Er(III). *J. Hazard. Mater.* **2009,** *170*, 1125-1132 https://doi.org/10.1016/j.jhazmat.2009.05.089
51. Li, L.; Liu, F.; Jing, X.; Ling, P.; Li, A., Displacement mechanism of binary competitive adsorption for aqueous divalent metal ions onto a novel IDA-chelating resin: isotherm and kinetic modeling. *Water Res.* **2011,** *45*, 1177-1188 https://doi.org/10.1016/j.watres.2010.11.009
52. Rengaraj, S.; Yeon, K.-H.; Kang, S.-Y.; Lee, J.-U.; Kim, K.-W.; Moon, S.-H., Studies on adsorptive removal of Co(II), Cr(III) and Ni(II) by IRN77 cation-exchange resin. *J. Hazard. Mater.* **2002,** *92*, 185-198 https://doi.org/10.1016/S0304-3894(02)00018-3
53. Atia, A. A.; Donia, A. M.; Yousif, A. M., Synthesis of amine and thio chelating resins and study of their interaction with zinc(II), cadmium(II) and mercury(II) ions in their aqueous solutions. *React. Funct. Polym.* **2003,** *56*, 75-82 https://doi.org/10.1016/S1381-5148(03)00046-4
54. Xu, Y.; Zeng, X.; Zhang, B.; Zhu, X.; Zhou, M.; Zou, R.; Sun, P.; Luo, G.; Yao, H., Experiment and kinetic study of elemental mercury adsorption over a novel chlorinated sorbent derived from coal and waste polyvinyl chloride. *Energy Fuels* **2016,** *30*, 10635-10642 https://doi.org/10.1021/acs.energyfuels.6b01372
55. Xiao, H.; He, B.; Qian, L.; Li, J., Cationic polystyrene spheres for removal of anionic contaminants in white water of papermaking. J. Appl. Polym. Sci. **2015,** *132* https://doi.org/10.1002/app.41379
56. Sun, C.; Qu, R.; Ji, C.; Wang, C.; Sun, Y.; Yue, Z.; Cheng, G., Preparation and adsorption properties of crosslinked polystyrene-supported low-generation diethanolamine-typed dendrimer for metal ions. *Talanta* **2006,** *70*, 14-19 https://doi.org/10.1016/j.talanta.2006.01.011
57. Li, J.-h.; Miao, X.-x.; Chen, X.-y.; Lu, L.; Yang, Y.; Fu, Y.-q.; Xiong, C.-h., Application and characterization of grafted polytetrafluoroethylene fiber for enhanced adsorption of Cu(II) in aqueous solutions. *J. Cent. South Univ.* **2016,** *23*, 2513-2519 https://doi.org/10.1007/s11771-016-3311-x
58. Navarro, R. R.; Sumi, K.; Fujii, N.; Matsumura, M., Mercury removal from wastewater using porous cellulose carrier modified with polyethyleneimine. *Water Res.* **1996,** *30*, 2488-2494 https://doi.org/10.1016/0043-1354(96)00143-1
59. Kang, S. Y.; Lee, J. U.; Moon, S. H.; Kim, K. W., Competitive adsorption characteristics of Co^{2+}, Ni^{2+}, and Cr^{3+} by IRN-77 cation exchange resin in synthesized wastewater. *Chemosphere* **2004,** *56*, 141-147 https://doi.org/10.1016/j.chemosphere.2004.02.004
60. Ji, C.; Song, S.; Wang, C.; Sun, C.; Qu, R.; Wang, C.; Chen, H., Preparation and adsorption properties of chelating resins containing 3-aminopyridine and hydrophilic spacer arm for Hg(II). *Chem. Eng. J.* **2010,** *165*, 573-580 https://doi.org/10.1016/j.cej.2010.09.075
61. Ho, W.-K.; Leung, K. S.-Y., The crucial role of heavy metals on the interaction of engineered nanoparticles with polystyrene microplastics. *Water Res.* **2021,** *201*, 117317 https://doi.org/10.1016/j.watres.2021.117317
62. Park, S. W.; Bediako, J. K.; Song, M.-H.; Choi, J.-W.; Lee, H.-C.; Yun, Y.-S., Facile fabrication of polyacrylic acid-polyvinyl chloride composite adsorbents for the treatment of cadmium-contaminated wastewater. *J. Environ. Eng.* **2018,** *6*, 2401-2408 https://doi.org/10.1016/j.jece.2018.03.038
63. Lang, M.; Yu, X.; Liu, J.; Xia, T.; Wang, T.; Jia, H.; Guo, X., Fenton aging significantly affects the heavy metal adsorption capacity of polystyrene microplastics. *Sci. Total Environ.* **2020,** *722*, 137762 https://doi.org/10.1016/j.scitotenv.2020.137762
64. Chen, C.; Chen, L.; Yao, Y.; Artigas, F.; Huang, Q.; Zhang, W., Organotin release from polyvinyl chloride microplastics and concurrent photodegradation in water: Impacts from salinity, dissolved organic matter, and light exposure. *Environ. Sci. Technol.* **2019,** *53*, 10741-10752 https://doi.org/10.1021/acs.est.9b03428
65. Mahanta, D.; Madras, G.; Radhakrishnan, S.; Patil, S., Adsorption and desorption kinetics of anionic dyes on doped polyaniline. *J. Phys. Chem. B* **2009,** *113*, 2293-2299 https://doi.org/10.1021/jp809796e
66. Nath, K.; Maity, K.; Biradha, K., Two-dimensional coordination polymers with "X"-Shaped cavities as adsorbents of oxoanion pollutants and toxic dyes. *Cryst. Growth Des.* **2017,** *17*, 4437-4444 https://doi.org/10.1021/acs.cgd.7b00771
67. Qu, R.; Wang, C.; Ji, C.; Sun, C.; Sun, X.; Cheng, G., Preparation, characterization, and metal binding behavior of novel chelating resins containing sulfur and polyamine. J. Appl. Polym. Sci. 2005, 95, 1558-1565 https://doi.org/10.1002/app.21370
68. Deng, S.; Bai, R.; Chen, J. P., Aminated polyacrylonitrile fibers for lead and copper removal. *Langmuir* **2003,** *19*, 5058-5064 https://doi.org/10.1021/la034061x
69. Dong, Y.; Gao, M.; Qiu, W.; Song, Z., Adsorption of arsenite to polystyrene microplastics in the presence of humus. *Environmental Science: Processes & Impacts* **2020,** *22*, 2388-2397 https://doi.org/10.1039/D0EM00324G

70. Yu, C.; Lin, W.; Jiang, J.; Jing, Z.; Hong, P.; Li, Y., Preparation of a porous superhydrophobic foam from waste plastic and its application for oil spill cleanup. *RSC Adv.* **2019,** *9*, 37759-37767 https://doi.org/ 10.1039/C9RA06848A
71. Gong, J.; Liu, J.; Chen, X.; Jiang, Z.; Wen, X.; Mijowska, E.; Tang, T., Striking influence of NiO catalyst diameter on the carbonization of polypropylene into carbon nanomaterials and their high performance in the adsorption of oils. *RSC Adv.* **2014,** *4*, 33806-33814 https://doi.org/10.1039/C4RA05016A
72. Hinrichs, R.; Snoeyink, V., Sorption of benzenesulfonates by weak base anion exchange resins. *Water Res.* **1976,** *10*, 79-87 https://doi.org/10.1016/0043-1354(76)90161-5
73. Lhoest, J. B.; Detrait, E.; Van Den Bosch De Aguilar, P.; Bertrand, P., Fibronectin adsorption, conformation, and orientation on polystyrene substrates studied by radiolabeling, XPS, and ToF SIMS. *J. Biomed. Mater. Res.* **1998,** *41*, 95-103 https://doi.org/10.1002/(SICI)1097-4636(199807)41:1<95::AID-JBM12>3.0.CO;2-G
74. Boisson, C.; Jozefonvicz, J.; Brash, J. L., Adsorption of thrombin from buffer and modified plasma to polystyrene resins containing sulphonate and sulphamide arginyl methyl ester groups. *Biomaterials* **1988,** *9*, 47-52 https://doi.org/10.1016/0142-9612(88)90068-3
75. Qian, D.; Bai, L.; Wang, Y.-S.; Song, F.; Wang, X.-L.; Wang, Y.-Z., A bifunctional alginate-based composite hydrogel with synergistic pollutant adsorption and photocatalytic degradation performance. *Ind. Eng. Chem. Res.* **2019,** *58,* 13133-13144 https://doi.org/10.1021/acs.iecr.9b01709
76. Chao, Y.; Zhu, W.; Ye, Z.; Wu, P.; Wei, N.; Wu, X.; Li, H., Preparation of metal ions impregnated polystyrene resins for adsorption of antibiotics contaminants in aquatic environment. *J. Appl. Polym. Sci.* **2015,** *132* https://doi.org/10.1002/app.41803
77. Zhu, H.; Qiu, S.; Jiang, W.; Wu, D.; Zhang, C., Evaluation of electrospun polyvinyl chloride/polystyrene fibers as sorbent materials for oil spill cleanup. *Environ. Sci. Technol.* **2011,** *45,* 4527-4531 https://doi.org/10.1021/es2002343
78. Guo, N. N.; Zhang, Q. G.; Li, H. M.; Wu, X. M.; Liu, Q. L.; Zhu, A. M., Facile fabrication, structure, and applications of polyvinyl chloride mesoporous membranes. *Ind. Eng. Chem. Res.* **2014,** *53*, 20068-20073 https://doi.org/10.1002/jctb.2089
79. Liu, P.; Lu, K.; Li, J.; Wu, X.; Qian, L.; Wang, M.; Gao, S., Effect of aging on adsorption behavior of polystyrene microplastics for pharmaceuticals: Adsorption mechanism and role of aging intermediates. *J. Hazard. Mater.* **2020,** *384*, 121193 https://doi.org/10.1016/j.jhazmat.2019.121193
80. Zhang, B.; Yu, S.; Zhu, Y.; Shen, Y.; Gao, X.; Shi, W.; Tay, J. H., Adsorption mechanisms of crude oil onto polytetrafluoroethylene membrane: Kinetics and isotherm, and strategies for adsorption fouling control. *Sep. Purif. Technol.* **2020,** *235*, 116212 https://doi.org/10.1016/j.seppur.2019.116212
81. Soetaredjo, F. E.; Ismadji, S.; Foe, K.; Woworuntu, G. L., Removal of Hazardous contaminants from water or wastewater using polymer nanocomposites Materials. *Nanotechnology for Sustainable Water Resources:* **2018**，*2*，103-139 https://doi.org/10.1002/9781119323655.ch4
82. Yang, L.; Zhang, H.; Tan, T.; Rahman, A. U., Thermodynamic and NMR investigations on the adsorption mechanism of puerarin with oligo-β-cyclodextrin-coupled polystyrene-based matrix. *J. Chem. Technol. Biotechnol.* **2009,** *84,* 611-617 https://doi.org/10.1002/jctb.2089
